# Efficacy and safety of Lenvatinib-based combination therapies for patients with unresectable hepatocellular carcinoma: a single center retrospective study

**DOI:** 10.3389/fimmu.2023.1198562

**Published:** 2023-07-07

**Authors:** Jian Huang, Zhen-Guang Wang, Qi-Fei Tao, Yun Yang, Sheng-Xian Yuan, Fang-Ming Gu, Hui Liu, Ze-Ya Pan, Bei-Ge Jiang, Wan Yee Lau, Wei-Ping Zhou

**Affiliations:** ^1^The Third Department of Hepatic Surgery, Eastern Hepatobiliary Surgery Hospital, Third Affiliated Hospital, Naval Medical University, Shanghai, China; ^2^Faculty of Medicine, The Chinese University of Hong Kong, Hong Kong, Hong Kong SAR, China

**Keywords:** hepatocellular carcinoma, Lenvatinib, combination therapy, PD-1, tumor-downstaging

## Abstract

**Background:**

Reports on Lenvatinib-based therapies show promising treatment outcomes for patients with unresectable hepatocellular carcinoma (uHCC). However, the effect and safety of Lenvatinib-based therapies still need to be further studies.

**Methods:**

This was a retrospective, single-center study on the safety and treatment efficacy of Lenvatinib-based combination therapies for uHCC Patients. The primary endpoints were progression-free survival (PFS) and overall survival (OS). The secondary endpoints were progressive disease (PD), stable disease (SD), partial response (PR), and complete response (CR).

**Results:**

Of 91 patients, there were 16 females and 75 males with uHCC who received systemic therapies based on Lenvatinib in our center. Forty-six patients (50.5%) received Lenvatinib combined with PD-1 antibody treatment. All these patients also received local therapy with the exception of 2 patients. The remaining 36 patinets received Lenvatinib combined with transcatheter arterial chemoembolization (TACE), 1 patient treated Lenvatinib combined with radiotherapy, 8 patients received Lenvatinib alone. At a median treatment time of 8 months, the objective response rate (ORR) of the entire cohort was 58.2% (53 patients), including 7 patients with CR and 46 patients with PR. 21 patients (23.1%) had SD. The disease control rate (DCR) of all patients was 81.3% (74 patients). However, 17 patients (18.7%) developed PD. The 1- and 2-year cumulative OS rates for the entire cohort were 66.8% and 39.3%, while the corresponding PFS rates were 38.0% and 17.1%, respectively. Univariate and multivariate Cox regression analysis revealed multiple tumor sites to be an independent OS risk factor for uHCC patients (HR=2.204, 95% CI=1.104-4.399, *P*=0.025). The most frequently reported adverse events in all patients were AST elevation (51.6%), followed by hypertension (33.0%), ALT elevation (26.4%), and decreased appetite (25.3%). After a combination treatment of Lenvatinib-based therapies, 15 patients met the criteria for salvage liver resection and underwent down-staging hepatectomy with a curative intent. The combination of PD-1 treatment was not very effective in improving the prognosis of uHCC patients treated with Lenvatinib combined with TACE.

**Conclusion:**

Our study demonstrated that a proportive of patients benefited from Lenvatinib-based combination therapies with manageable safety profiles, allowing these patients to undergo downstaging surgery with curative intent.

## Introduction

Hepatocellular carcinoma (HCC) accounts for 75-85% of all liver cancers and is the fourth leading cause of cancer-related mortality worldwide (1). The vast majority of cases of HCC are associated with chronic liver diseases such as chronic viral hepatitis and nonalcoholic fatty liver disease (2). Patients with unresectable HCC (uHCC) have an abysmal prognosis, and the late diagnosis is exacerbated by the absence of sysptoms and the paucity of effective screening programs for early diagnosis (3). Constraints for surgical resection as a common tool used for “care” come from factors such as chronic cirrhosis, large tumor size, and extrahepatic metastases. Systemic therapy is commonly used in patients with uHCC to prolong survival, enhance quality of life, and provide improved treatment outcomes.

Tyrosine kinase inhibitors (TKIs) are often used to treat uHCC. Lenvatinib, functioning as a multiple kinase inhibitor against the FGFR 1-4, VEGFR 1-3, RET, PDGF receptor α, and KIT, has been approved as the first-line systemic therapy drug for uHCC (1). The overall survival (OS) for advanced HCC patients treated with Lenvatinib has been shown to be non-inferior to sorafenib in the phase 3 clinical trial ‘REFLECT’ (2). The overall response rate (ORR) in the study on Lenvatinib in treating uHCC using the modified Response Evaluation Criteria in Solid Tumors (mRECIST) criteria was 24.1%. However, 99% of patients with uHCC in this study developed treatment-emergent adverse events (TEAEs). Previous studies have also showed that combination treatments comprising of vascular endothelial growth factor (VEGF)-mediated immune suppression and PD-1 inhibitors, such as pembrolizumab, are promising options in achieving better prognosis for HCC patients, especially in HBV-related HCC (3, 4). The synergistic effect of the combinations lies in decreased number of tumor-associated macrophages and a high proportion of CD8+ T cells, both of which can increase the antitumor activity of PD-1 inhibitors (5). For patients with HCC in the intermediate and advanced stages, transcatheter arterial chemoembolization (TACE) has become the gold standard of treatment (6). It has been shown that TACE has a synergistic effect with systemic therapies because it upregulates the expressions of fibroblast growth factor (FGF) and VEGF (7), and stimulates tumor-associated antigens release and immunogenic death of cancer cells (8, 9). Other local treatments such as radiotherapy, radiofrequency ablation, and hepatic artery infusion chemotherapy (HAIC), also showed definite therapeutic effects on advanced HCC (10). uHCC with a large tumor size and rich blood supply can limit the treatment outcome of local treatments, making the need for them to be combined with other therapies to obtain satisfactory long-term survival results.

Whether or not combined therapies can be used to enhance treatment efficacy but without increasing adverse effects are the major concerns for clinicians. However, the benefits and drawbacks of using Lenvatinib-based combination treatments in HCC are still unclear. This retrospective study evaluated treatment efficacy and adverse effects of Lenvatinib-based combination therapies on uHCC patients treated in our center.

## Materials and methods

### Patients

Patients who wre included in this retrospective study came from the Eastern Hepatobiliary Surgery Hospital (EHBH) between December 2018 to October 2020. Data on clinical outcomes and histopathological findings were collected prospectively and examined retrospectively. The Declaration of Helsinki was followed, and the EHBH Hospital’s Ethics Committee approved this research. All participants provided written informed consent for the treatment and for their data to be used for clinical research.

All eligible patients were independently diagnosed and evaluated by two experienced pathologists using the Barcelona Clinic Liver Cancer (BCLC) staging (11) and the Child-Pugh classification. Based on the Liver Cancer Study Group of Japan, uHCC was defined as bi-lobar liver involvement due to multiple or large solitary tumors, insufficient future liver remnant, extrahepatic metastasis or major vessels invasion, including inferior vena cava and portal vein (12).

The inclusion criteria were patients with: (1) uHCC confirmed by clinical features or histopathological biopsy; (2) Eastern Cooperative Oncology Group (ECOG) performance status (PS) 0 or 1; (3) Child-Pugh class A or B; (4) at least one measurable target lesion by mRECIST. The exclusion criteria were patients with: (1) ECOG-PS score >1; (2) Child-Pugh class C; (3) a history of other cancers; (4) incomplete data. Patients with uHCC who simultaneously received other forms of therapy such as TACE, radiotherapy, radiofrequency ablation, or chemotherapy were also included in this study. Surgeons based on the relevant guidelines combined with patient’s general condition, tumor burden status, laboratory tests, imaging examination and other indicators to determine which treatments to receive.

### Data collection and definitions

Clinical characteristics of patients including sex, age, liver cirrhosis, albumin-bilirubin (ALBI) grading, liver function, hepatitis virus status and tumor biomarkers were collected before treatment. Tumor status, including extrahepatic metastases, macrovascular invasion, BCLC staging and CNLC staging (13) were determined based on the results of imaging examinations. We used the following formula to mathematically derive ALBI: (log 10 bilirubin (umol/L) x 0.66) + (albumin (g/L) x -0.085) (14). The threshold level of HBV-DNA was used as we have previously described (15).

### Treatment protocols

Lenvatinib (Eisai Inc., Woodcliff Lake, NJ, USA) was administered to all patients at a daily dose of 8 mg for individuals weighing <60 kg and 12 mg for those weighing ≥ 60 kg as administered in the REFLECT trial. All dose interruptions and reductions were carried out strictly using the REFLECT trial’s protocol. In most patients, Lenvatinib therapy was given for 8 months (range 5-11 months). Labeled patients were given 200 mg of pembrolizumab (Merck, New York, NJ) intravenously once every three weeks. Severe adverse events which warranted to discontinue treatment was adopted. The Seldinger technique was used to carry out TACE by catheterization of the hepatic artery through the percutaneous femoral artery to selectively or superselectively intubate the artery that supplies blood to the tumor. Proper amount of lipiodol and gelatin sponge, as well as the chemotherapeutic drugs of fluorouracil, epirubicin, and platinum were administered through the catheter. The chemotherapy medication doses were based on the patient’s tumor stage, body area, and physical condition.

CT or MRI was conducted to evaluate the treated tumor(s) once every 6-8 weeks, or when there were signs or symptoms indicating tumor progression. The RECIST v1.1 was used to grade tumor responses. Grading of AEs complied with CTCAE v5.0. Radiotherapy and radiofrequency ablation were performed based on the patient’s condition to achieve the best treatment effect.

### Evaluation indexes

The primary endpoints were progression free survival (PFS) and OS. PFS was defined as the period between the first day of Lenvatinib treatment and the date of disease progression was measured by RECIST v1.1 or death. OS was measured from the first day of Lenvatinib treatment to the date the patient died. The secondary endpoints were tumor response rates, including CR, PR, SD, and PD. The ORR was calculated as the sum of PR and CR, while the DCR was the sum of PR, CR, and SD. The treatment effect of each patient was evaluated by mRECIST based on contrast-enhanced CT or MRI. The AEs were graded using the NCI-CTCAE v5.0.

### Follow-up

Every patient was followed-up once every 6-8 weeks for monitoring and evaluation. Regular blood and urine tests, liver and kidney liver functions, thyroid function, myocardial enzymes, HBV-DNA, and tumor markers including Prothrombin caused by Vitamin K Absence or Antagonist-II (PIVKA-II) and alpha-fetoprotein (AFP) and CT or MRI to assess tumor response were performed at each follow-up visit. Additionally, routine chest X-rays were done. With patient’s informed consent, radical resection with postoperative adjuvant treatment were carried out after any patient who met the tumor-downstaging criteria for salvage resection. Lenvatinib was discontinued for 1 week, and PD-1 was discontinued for 1 month before and after surgery. The remaining patients were followed-up until they developed PD, with symptomatic progression, developed severe toxicity to drug treatment, or withdrew their consents.

### Statistical analysis

SPSS 26.0 (IBM, New York, United States) and Graph Pad Prism 9.0 (San Diego, CA, USA) were used for data processing and analysis. Data were presented as mean ± SD for continuous variables and as percentiles for categorical ones. Univariate and multivariate analysis were carried out using the Cox proportional hazards model. Fisher’s exact or Chi-squared tests were used for differences between categorical variables. Continuous variables were compared by the Student’s *t*-test or Mann-Whitney U test. The Kaplan-Meier technique was used for survival analysis and compared using the log-rank test. A *P*<0.05 was deemed statistically significant.

## Results

### Baseline characteristics of uHCC patients

This study included 91 patients with uHCC. There were 16 females and 75 males who received Lenvatinib-based systemic therapies between December 2018 and October 2020. The median age was 53 years (interquartile range, 46.0-58.0), and 78 patients (85.7%) were HBsAg-positive, 52 patients (57.1%) had liver cirrhosis, nearly all patients were in Child-Pugh class A, and only three patients (3.3%) in Child-Pugh class B. Tumor markers showed 64 patients (70.3%) to have AFP levels above 20μ g/L, and 76 patients (83.5%) had elevated PIVKA-II levels. The median tumor diameter was 6.0 cm (interquartile range, 4.0-9.0cm), and 58 patients (63.7%) had multiple tumors. Ten patients (11.0%) had lymph node metastasis, 21 patients (23.1%) had extrahepatic metastasis, and 29 patients (31.9%) had macrovascular invasion, which included portal vein and hepatic vein invasion. Tumor staging, showed 57.1% and 56.0% of patients were categorized as BCLC stage C, and China Live Cancer (CNLC) stage IIIa-IIIb, respectively. Specifically, among the BCLC stage A patients, three patients with tumor recurrence after surgery were reluctant to undergo further surgery, five patients had unresectable HCC due to large tumor sizes, and one patient received targeted therapy for multiple tumors including multiple liver resections. Notably, 15 patients who received the systemic treatment were down-staged to salvage liver resection aiming at clinical cure. **Table 1** provides the summary of the baseline characteristics of the patients.

**Table 1:** Clinical characteristics of patients with unresectable HCC.

Variables	Whole cohort (n=91)
Sex (Female/Male)	16 (17.6%)/75 (82.4%)
Age, year, median (Q1, Q3)	53.0 (46.0, 58.0)
Liver cirrhosis (No/Yes)	39 (42.9%)/52 (57.1%)
Child-Pugh (A/B)	88 (96.7%)/3 (3.3%)
ALBI, median (Q1, Q3)	-2.78 (-2.94, -2.55)
ALBI grade	
1	64 (70.3%)
2	27 (29.7%)
3	0 (0%)
HBsAg (Negative/Positive)	13 (14.3%)/78 (85.7%)
HBV-DNA load, IU/ml (≤2000/>2000)	60 (65.9%)/31 (34.1%)
Antivirus therapy (No/Yes)	49 (53.8%)/42 (46.2%)
AFP, μg/L (≤20/>20)	27 (29.7%)/64 (70.3%)
PIVKA-II, μg/L (≤37/>37)	15 (16.5%)/76 (83.5%)
CEA, μg/L (≤10/>10)	87 (95.6%)/4 (4.4%)
CA19-9, μg/L (≤39/>39)	71 (78.0%)/20 (22.0%)
Maximum tumor size, cm, median (Q1, Q3)	6.0 (4.0, 9.0)
Tumor number (1/2/3/≥4)	33 (36.3%)/27 (29.7%)/10 (11.0%)/21 (23.1%)
Lymph node metastasis (No/Yes)	81 (89.0%)/10 (11.0%)
Extrahepatic metastasis (No/Yes)	70 (76.9%)/21 (23.1%)
Macrovascular invasion	
No	62 (68.1%)
Portal vein	9 (9.9%)
Hepatic vein	16 (17.6)
Portal vein combined with hepatic vein	4 (4.4%)
Duration time, month, median (Q1, Q3)	8.0 (4.0, 12.0)
BCLC (A/B/C)	9 (9.9%)/30 (33.0%)/52 (57.1%)
CNLC stage (Ia-Ib/IIa-IIb/IIIa-IIIb)	13 (14.3%)/27 (29.7%)/51 (56.0%)

HCC, hepatocellular carcinoma; ALBI, Albumin-bilirubin grade; Q1, first quartile; Q3, third quartile; AFP, alpha-fetoprotein; PIVKA-II, Protein Induced by Vitamin K Absence or Antagonist-II; CA19-9, carbohydrate antigen 19-9; CEA, carcino-embryonic antigen; HBsAg, hepatitis B surface antigen; HBV-DNA, hepatitis B virus deoxyribonucleic acid; BCLC, Barcelona Clinic Liver Cancer; CNLC, China liver cancer staging.

### Systemic and local therapy regimen for uHCC

**Table 2** is a summary of the treatment regimens used in this study. Most patients were treated with systemic and local therapy, and only seven patients (7.6%) were treated with Lenvatinib alone. The majority of local therapy consisted of TACE, radiotherapy, radiofrequency ablation, and hepatic artery infusion chemotherapy (HAIC). TACE was administered to 78 patients (85.7%), of whom 44 patients (48.4%) were treated once, and 9 patients (9.8%) were treated for more than four times. Based on the patient’s condition,46 patients (50.5%) received Lenvatinib combined with PD-1 antibody therapy, with only 2 patients who received this combination treatment alone while the remainding patients (n=44) received this combination treatment together with local therapy. Eighty-two patients (90.1%) received 8 mg/day of Lenvatinib, while 9 (9.9%) received 12 mg/day.

**Table 2:** Specific treatment regiments for patients with advanced unresectable HCC.

Treatment regiments	n=91
TACE	
0	13 (14.3%)
1	44 (48.4%)
2	15 (16.5%)
3	10 (11.0%)
≥4	9 (9.8%)
Lenvatinib dose (mg)	
8	82 (90.1%)
12	9 (9.9%)
Lenvatinib combined with PD-1	
No	44 (48.4%)
Yes	47 (51.6%)
Lenvatinib combined with other therapies	
Lenvatinib	8 (8.7%)
Lenvatinib + PD-1	2 (2.2%)
Lenvatinib + PD-1+ radiotherapy	2 (2.2%)
Lenvatinib + TACE	22 (24.2%)
Lenvatinib + TACE + PD-1	20 (22.0%)
Lenvatinib + TACE + PD-1 + radiotherapy	14 (15.4%)
Lenvatinib + TACE + radiotherapy	9 (9.9%)
Lenvatinib + TACE + radio frequency	4 (4.4%)
Lenvatinib + TACE + PD-1+radio frequency	6 (6.6%)
Lenvatinib + TACE + PD-1+radio frequency+radiotherapy	1 (1.1%)
Lenvatinib + TACE + PD-1+radio frequency+radiotherapy+HAIC	1 (1.1%)
Lenvatinib + TACE + radio frequency+radiotherapy	1 (1.1%)
Lenvatinib + radiotherapy	1 (1.1%)

TACE, transcatheter arterial chemoembolization; PD-1, PD-1 antibody; HAIC, Hepatic artery infusion chemotherapy.

### Efficacy and prognosis after treatment

The ORR of the entire cohort was 58.2% (n=53) after a median treatment of 8 months, there were 7 patients with CR and 46 patients with PR. In addition 21 patients (23.1%) achieved SD. The DCR for all the patients was 81.3% (n=74). The remaining 17 patients (18.7%) had PD. **Table 3** summarizes the response rates as stratified by the BCLC staging.

**Table 3:** Best tumor response in all patients and subgroups.

	Total n=91	BCLC stage A n=9	BCLC stage B n=30	BCLC stage C n=52
CR	7 (7.7%)	2 (22.2%)	1 (3.3%)	4 (7.7%)
PR	46 (50.5%)	6 (66.7%)	14 (46.7%)	26 (50.0%)
SD	21 (23.1%)	1 (11.1%)	6 (20.0%)	10 (19.2%)
PD	17 (18.7%)	0 (0)	9 (30.0%)	12 (23.1%)
ORR*	53 (58.2%)	8 (88.9%)	15 (50.0%)	30 (57.7%)
DCR	74 (81.3%)	9 (100%)	21 (70.0%)	40 (76.9%)
Median OS (m)	17	/	17	17
Median PFS (m)	10	23.5	11	10

CR, complete response; PR, partial response; SD, stable disease; PD, progressive disease; ORR, objective response rate; DCR, disease control rate; OS, overall survival; PFS, progression-free survival.

*The P value of ORR between BCLC stage A, B and C was 0.084; between BCLC stage A and B was 0.09; between BCLC stage A and C was 0.158; between BCLC stage B and C was 0.500.

**Figure 1:**
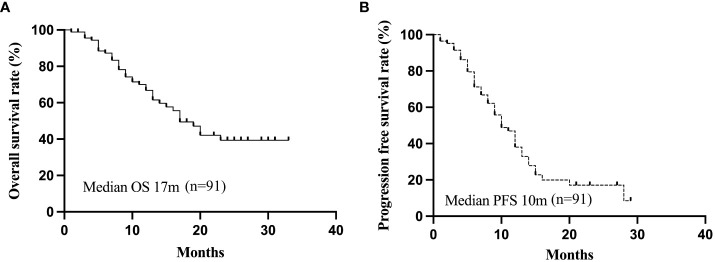
The all-patients Kaplan-Meier curves for OS **(A)** and PFS **(B)**. OS, overall survival; PFS, progression-free survival.

**Figure 2:**
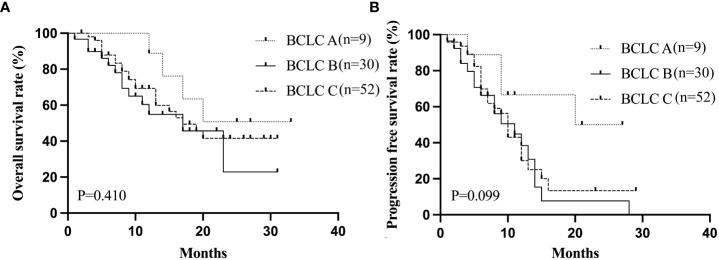
The Kaplan-Meier curves for OS **(A)** and PFS **(B)** stratified by BCLC staging. OS, overall survival; PFS, progression-free survival.

The patients were observed for a duration ranging from 4-37 months (median 21 months). The 1- and 2-year cumulative OS rates for the entire cohort were 66.8% and 39.3%, while the corresponding PFS rates were 38.0% and 17.7%, respectively. The median PFS and OS were 10 and 17 months, respectively (**Figure 1**). On stratification using the BCLC staging, the median OS were 17 months for both the BCLC stages B and C. (**Figure 2A**). The median PFS for the BCLC stages A, B, and C were 23.5, 11, and 10 months, respectively (**Figure 2B**). Univariate and multivariate Cox regression analyses on the entire patient cohort for prognosis revealed that multiple tumor sites to be an independent risk factor for OS in uHCC patients (HR=2.204, 95% CI=1.104-4.399, P=0.025) (**Table 4**). **Table 5** shows 11 patients received alternative therapies following progression of the tumors.

**Table 4:** Univariate and multivariate analysis of OS and PFS for patients with unresectable HCC.

Variables	OS			PFS		
	HR	95%CI	*P* value	HR	95%CI	*P* value
Univariate analysis						
Sex (Female/Male)	1.395	0.610-3.189	0.430	1.326	0.665-2.645	0.423
Age, year, (≤60, >60)	1.964	0.939-4.109	0.073	0.746	0.375-1.485	0.404
ALBI grade (1/2)	1.615	0.831-3.139	0.158	1.241	0.705-2.184	0.454
Antivirus therapy (No/Yes)	0.906	0.478-1.716	0.762	1.390	0.816-2.366	0.225
CA19-9, μg/L (≤39/>39)	0.824	0.363-1.869	0.643	1.049	0.540-2.037	0.887
CEA, μg/L (≤10/>10)	0.927	0.222-3.870	0.917	0.657	0.159-2.706	0.561
AFP, μg/L (≤20/>20)	1.931	0.913-4.081	0.085	1.130	0.645-1.979	0.670
PIVKA-II, μg/L (≤37/>37)	1.009	0.444-2.292	0.984	0.798	0.417-1.527	0.495
Child-Pugh (A/B)	3.099	0.947-10.139	0.061	1.603	0.498-5.161	0.429
HBsAg (Negative/Positive)	0.842	0.353-2.011	0.699	1.151	0.540-2.452	0.715
HBV-DNA load, IU/ml (≤2000/>2000)	0.495	0.227-1.076	0.076	0.722	0.408-1.277	0.263
Liver cirrhosis (No/Yes)	1.144	0.604-2.168	0.680	1.355	0.787-2.331	0.273
Extrahepatic metastasis (No/Yes)	0.741	0.358-1.536	0.421	1.036	0.563-1.906	0.910
Lymph node metastasis (No/Yes)	0.509	0.156-1.655	0.261	1.154	0.519-2.562	0.726
Macr0vascular invasion (No/Yes)	1.091	0.522-2.280	0.817	1.028	0.556-1.899	0.931
Tumor number (Single/Multiple)	2.204	1.104-4.399	***0.025***	1.595	0.910-2.795	0.103
Maximum tumor size, cm, (≤3, 3~5, >5)	1.519	0.981-2.354	0.061	1.140	0.813-1.600	0.447
Combined with PD-1(No/Yes)	0.911	0.483-1.720	0.774	1.189	0.693-2.039	0.530
Combined with radiotherapy (No/Yes)	0.700	0.341-1.436	0.331	0.677	0.374-1.227	0.199
Combined with TACE (No/Yes)	1.096	0.457-2.628	0.837	1.147	0.538-2.443	0.723
Lenvatinib ± PD-1vs. lenvatinib ± PD-1+ TACE ± radiotherapy	1.065	0.646-1.757	0.805	0.705	0.476-1.045	0.082
BCLC (A/B/C)	1.102	0.717-1.693	0.657	1.338	0.916-1.955	0.132
CNLC stage (Ia-Ib/IIa-IIb/IIIa-IIIb)	1.052	0.700-1.582	0.806	1.240	0.864-1.780	0.243
Multivariate analysis						
Tumor number (Single/Multiple)	2.204	1.104-4.399	***0.025***			

HCC, hepatocellular carcinoma; OS, overall survival; PFS, progression-free survival; ALBI, Albumin-bilirubin grade; AFP, alpha-fetoprotein; PIVKA-II, Protein Induced by Vitamin K Absence or Antagonist-II; CA19-9, carbohydrate antigen 19-9; CEA, carcino-embryonic antigen; HBsAg, hepatitis B surface antigen; HBV-DNA, hepatitis B virus deoxyribonucleic acid; BCLC, Barcelona Clinic Liver Cancer; CNLC, China liver cancer staging; TACE, transcatheter arterial chemoembolization. P<0.05 was defined as statistical significance (italicized and bold).

**Table 5:** Alternative treatment options for patients with advanced unresectable HCC after tumor progression.

Treatment options	n=11
Replacement targeted agents	
Regorafenib	3
Apatinib	4
Attilizumab + bevacizumab	2
Add other local treatment	
TACE	8
Cyberknife	2

HCC, hepatocellular carcinoma; TACE, transcatheter arterial chemoembolization.

### Adverse events

In this study, all AEs were assessed and found to be tolerable and mild, and there were no toxicity-related deaths. The AEs in patients consisted of elevated AST levels (51.6%), followed by hypertension (33%), elevated ALT levels (26.4%), and decreased appetite (25.3%). AEs of grade 3 or higher were experienced in 24.2% of all patients to include AST elevation (6.6%), hypertension (4.4%), diarrhea (4.4%), increased blood bilirubin (4.4%), decreased appetite (3.3%), and fatigue (1.1%) (**Table 6**).

**Table 6:** Adverse events.

	All (n=91)	
Symptoms	Any grade	Grade 3-4
Any adverse event	88 (96.7%)	22 (24.2%)
Fatigue	10 (11.0%)	1 (1.1%)
Hypertension	30 (33.0%)	4 (4.4%)
Decreased appetite	23 (25.3%)	3 (3.3%)
Diarrhea	12 (13.1%)	4 (4.4%)
Hypothyroidism	4 (4.4%)	0
Proteinuria	3 (3.3%)	0
Dysphonia	2 (2.2%)	0
Epistaxis	2 (2.2%)	0
Fever	5 (5.5%)	0
Edema	3 (3.3%)	0
Cramps	3 (3.3%)	0
Nausea	13 (14.3%)	0
Vomiting	6 (6.6%)	0
Pneumonia	1 (1.1%)	0
Abdominal distension	2 (2.2%)	0
Hand-foot skin reaction	3 (3.3%)	0
Increased blood bilirubin	22 (24.2%)	4 (4.4%)
Rash	6 (6.6%)	0
Abdominal pain	2 (2.2%)	0
Increased creatinine	1 (1.1%)	0
ALT elevation	24 (26.4%)	0
AST elevation	47 (51.6%)	6 (6.6%)

ALT, alanine transaminase; AST, aspartate aminotransferase.

### Tumor-downstaging followed by salvage liver resection

**Figure 3:**
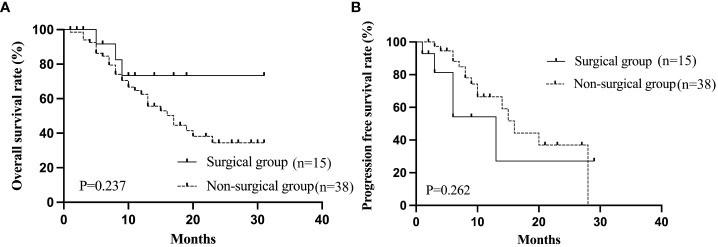
The Kaplan-Meier curves of OS **(A)** and PFS **(B)** stratified by surgery in patients who achieved ORR. OS, overall survival; PFS, progression-free survival; ORR, objective response rate.

Following Lenvatinib-based combination therapies, the tumors in 15 patients were down-staged to become resectable using salvage liver resection with a curative intent. Seven (23.3%) of these 15 patients who received down-staging resection initially had BCLC stage B disease, while the remaining 8 patients (15.4%) initially had BCLC stage C disease. (**Table 7**). The baseline clinicopathologic characteristics of uHCC patients who achieved ORR after treatment as stratified by surgery are shown in **Table 8**, with no significantly differences in these characteristics which existed between the surgical and non-surgical groups. The 1- year cumulative OS and PFS rates for the surgical group were 73.3% and 27.7%, compared with the non-surgical group of 62.6% and 66.5%, respectively. The 2- year cumulative OS and PFS for the surgical group was not reached, while the non-surgical group was 34.4% and 36.4%.The median PFS of the surgical group was 13 months, compared with the non-surgical group of 16 months. The median OS of the surgical group was not reached, while the non-surgical group was 17 months. The two groups had no significant differences in OS (P=0.237) and PFS (P=0.262). **Figure 3** shows the Kaplan-Meier curves of OS and PFS for patients who achieved ORR as stratified by surgery.

**Table 7:** Number of patients treated after tumor down-staging with salvage livers resection.

The patients of down-stage surgery stratified by BCLC stage	n=15
BCLC stage A (N=9)	0
BCLC stage B (N=30)	7 (23.3%)
BCLC stage C (N=52)	8 (15.4%)

**Table 8:** Clinical characteristics of the surgical group that achieved ORR after treatment.

Variables	Surgical group	Non-surgical group	P value
	n=15	n=38	
Sex (Female/Male)	1 (6.7%)/14 (93.3)	8 (21.1%)/30 (78.9%)	0.395
Age, year, median (Q1, Q3)	48 (44, 57)	54 (46,59.3)	0.506
Liver cirrhosis (No/Yes)	4 (26.7%)/11 (73.3%)	18 (47.4%)/20 (52.6%)	0.285
Child-Pugh (A/B)	15 (100%)/0 (0%)	37 (97.4%)/1 (2.6%)	1.000
ALBI, median (Q1, Q3)	-2.81 (-2.83, -2.57)	-2.76 (-2.90, -2.49)	0.380
ALBI grade (1/2)	11 (73.3%)/4 (26.7%)	25 (65.8%)/13 (34.2%)	0.839
HBsAg (Negative/Positive)	2 (13.3%)/13 (86.7%)	5 (13.2%)/33 (86.8%)	1.000
HBV-DNA load, IU/ml (≤2000/>2000)	10 (66.7%)/5 (33.3%)	23 (60.5%)/15 (39.5%)	0.920
Antivirus therapy (No/Yes)	6 (40.0%)/9 (60.0%)	23 (60.5%)/15 (39.5%)	0.296
AFP, μg/L (≤20/>20)	5 (33.3%)/10 (66.7%)	11 (28.9%)/27 (71.1%)	1.000
PIVKA-II, μg/L (≤37/>37)	4 (26.7%)/11 (73.3%)	7 (18.4%)/31 (81.6%)	0.771
CEA, μg/L (≤10/>10)	14 (93.3%)/1 (6.7%)	37 (97.4%)/1 (2.6%)	1.000
CA19-9, μg/L (≤39/>39)	14 (93.3%)/1 (6.7%)	27 (71.1%)/11 (28.9%)	0.167
Maximun tumor size, cm, median (Q1, Q3)	5.0 (3.5, 12.6)	6.2 (3.8, 9.7)	0.837
Tumor number (1/2/3/≥4)	4 (26.7%)/7 (46.7%)/0 (0%)/4 (26.7%)	19 (50.0%)/7 (18.4%)/3 (7.9%)/9 (23.7%)	0.121
Tumor number (Single/Multiple)	4 (26.7%)/11 (73.3%)	19 (50.0%)/19 (50.0%)	0.216
Lymph node metastasis (No/Yes)	12 (80.0%)/3 (20.0%)	35 (92.1%)/3 (7.9%)	0.440
Extrahepatic metastasis (No/Yes)	13 (86.7%)/2 (13.3%)	31 (81.6%)/7 (18.4%)	0.969
Marcovascular invasion			
No	10 (66.7%)	24 (63.2%)	0.838
Portal vein	1 (6.7%)	6 (15.8%)	
Hepatic vein	3 (20.0%)	6 (15.8%)	
Portal vein combined with hepatic vein	1 (6.7%)	2 (5.3%)	
Marcovascular invasion (No/Yes)	10 (66.7%)/5 (33.3%)	24 (63.2%)/14 (36.8%)	1.000
Duration time, month, median (Q1, Q3)	7.0 (2.0, 10.0)	11.0 (6.0, 14.5)	0.069
Combined with PD-1 (No/Yes)	9 (60.0%)/6 (40.0%)	17 (44.7%)/21 (55.3%)	0.486
Combined with radiotherapy (No/Yes)	10 (66.7%)/5 (33.3%)	20 (52.6%)/18 (47.4%)	0.535
Combined with TACE (No/Yes)	2 (13.3%)/13 (86.7%)	5 (13.2%)/33 (86.8%)	1.000
BCLC (A/B/C)	0 (0%)/7 (46.7%)/8 (53.3%)	8 (21.1%)/8 (21.1%)/22 (57.9%)	0.058
CNLC stage (Ia-Ib/IIa-IIb/IIIa-IIIb)	2 (13.3%)/5 (33.3%)/8 (53.3%)	9 (23.7%)/8 (21.1%)/21 (55.3%)	0.542

HCC, hepatocellular carcinoma; Q1, first quartile; Q3, third quartile; AFP, alpha-fetoprotein; PIVKA-II, Protein Induced by Vitamin K Absence or Antagonist-II; CA19-9, carbohydrate antigen 19-9; CEA, carcino-embryonic antigen; HBsAg, hepatitis B surface antigen; HBV-DNA, hepatitis B virus deoxyribonucleic acid; BCLC, Barcelona Clinic Liver Cancer; CNLC, China liver cancer staging.

### The impact of PD-1 treatment in uHCC patients treated with Lenvatinib and TACE

**Figure 4:**
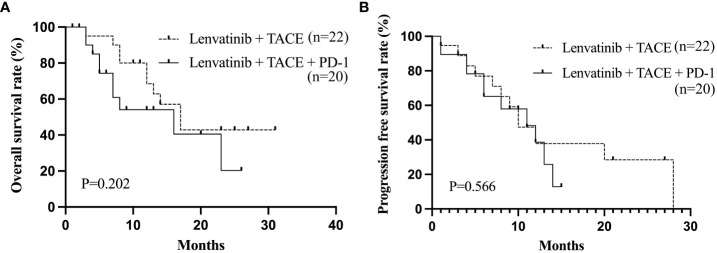
The Kaplan-Meier curves of OS **(A)** and PFS **(B)** of uHCC patients who achieved ORR after Lenvatinib and TACE treatment. OS, overall survival; PFS, progression-free survival; ORR, objective response rate; uHCC, unresectable hepatocellular carcinoma.

**Figure 5:**
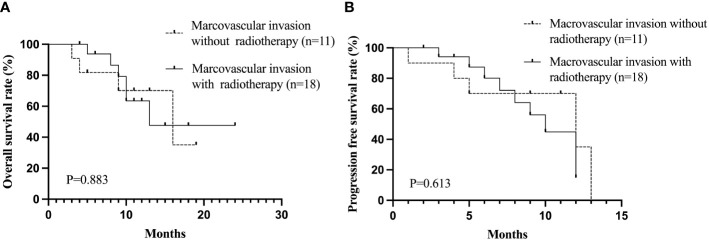
The Kaplan-Meier curves of OS **(A)** and PFS **(B)** of uHCC patients with macrovascular invasion with or without radiotherapy. OS, overall survival; PFS, progression-free survival; uHCC, unresectable hepatocellular carcinoma.

The baseline clinical characteristics of uHCC patients who achieved ORR following treatment with Lenvatinib and TACE are shown in **Table 9**. Patients (n=42) were divided into two groups based on the PD-1 treatment. The proportion of patients with macrovascular invasion was greater in the Lenvatinib + TACE + PD-1 group (n=20) than in the Lenvatinib + TACE group (n=22, P=0.034). The median OS and PFS were 16 months and 11 months in the Lenvatinib + TACE + PD-1 group and 17 months and 10 months in the Lenvatinib + TACE group, respectively. **Figure 4** depicts the Kaplan-Meier curves for OS (P=0.202) and PFS (P=0.566) for these patients. As the proportions of macrovascular invasion were significantly different between the Lenvatinib + TACE + PD-1 group and the Lenvatinib + TACE group, the effect of radiotherapy on patients with macrovascular invasion was also evaluated. **Figure 5** depicts the Kaplan-Meier curves for OS (P=0.883) and PFS (P=0.613) in patients with macrovascular invasion, with or without radiotherapy. The median OS and PFS of the macrovascular invasion group were 13 months and 10 months in the radiotherapy group compared with 16 months and 12 months in the group without radiotherapy, respectively.

**Table 9:** Clinical characteristics of patients who achieved ORR after Lenvatinib and TACE treatment.

Variables	Lenvatinib+TACE	Lenvatinib+TACE+PD-1	P value
	n=22	n=20	
Sex (Female/Male)	2 (9.1%)/20 (90.9%)	5 (25.0%)/15 (75.0%)	0.333
Age, year, median (Q1,Q3)	53 (43.0, 57.3)	54 (48.3, 63.3)	0.174
Liver cirrhosis (No/Yes)	8 (36.4%)/14 (63.6%)	8 (40.0%)/12 (60.0%)	1.000
ALBI, median (Q1,Q3)	-2.7 (-2.9, -2.5)	-2.7 (-3.1, -2.6)	0.508
ALBI grade (1/2)	14 (63.6%)/8 (36.4%)	15 (75.0%)/5 (25.0%)	0.644
HBsAg (Negative/Positive)	1 (4.5%)/21 (95.5%)	4 (20.0%)/16 (80.0%)	0.286
HBV-DNA load, IU/ml (≤2000/>2000)	15 (68.2%)/7 (31.8%)	13 (65.0%)/7 (35.0%)	1.000
Antivirus therapy (No/Yes)	9 (40.9%)/13 (59.1%)	15 (75.0%)/5 (25.0%)	0.055
AFP, μg/L (≤20/>20)	6 (27.3%)/16 (72.7%)	6 (30.0%)/14 (70.0%)	1.000
PIVKA-II, μg/L (≤37/>37)	4 (18.2%)/18 (81.8%)	2 (10.0%)/18 (90.0%)	0.753
CEA, μg/L (≤10/>10)	21 (95.5%)/1 (4.5%)	19 (95.0%)/1 (5.0%)	1.000
CA19-9, μg/L (≤39/>39)	16 (72.7%)/6 (27.3%)	15 (75.0%)/5 (25.0%)	1.000
Maximun tumor size, cm, median (Q1, Q3)	6 (3, 10.3)	6.8 (3.4, 9.6)	0.690
Tumor number (Single/Multiple)	9 (40.9%)/13 (59.1%)	3 (15.0%)/17 (85.0%)	0.130
Lymph node metastasis (No/Yes)	20 (90.9%)/2 (9.1%)	20 (100.0%)/0 (0%)	0.512
Extrahepatic metastasis (No/Yes)	16 (72.7%)/6 (27.3%)	16 (80.0%)/4 (20.0%)	0.849
Macrovascular invasion (No/Yes)	21 (95.5%)/1 (4.5%)	13 (65.0%)/7 (35.0%)	***0.034***
Duration time, month, median (Q1, Q3)	10.5 (5, 16)	6 (4.0, 9.8)	0.070
BCLC (A/B/C)	5 (22.7%)/9 (40.9%)/8 (36.4%)	1 (5.0%)/8 (40.0%)/11 (55.0%)	0.211
CNLC stage (Ia-Ib/IIa-IIb/IIIa-IIIb)	7 (31.8%)/7 (31.8%)/8 (36.4%)	1 (5.0%)/9 (45.0%)/10 (50.0%)	0.087

HCC, hepatocellular carcinoma; Q1, first quartile; Q3, third quartile; AFP, alpha-fetoprotein; PIVKA-II, Protein Induced by Vitamin K Absence or Antagonist-II; CA19-9, carbohydrate antigen 19-9; CEA, carcino-embryonic antigen; HBsAg, hepatitis B surface antigen; HBV-DNA, hepatitis B virus deoxyribonucleic acid; BCLC, Barcelona Clinic Liver Cancer; CNLC, China liver cancer staging. P<0.05 was defined as statistical significance (italicized and bold).

## Discussion

Our study supports that Lenvatinib-based combination therapies for patients with uHCC to be efficacious and safe, with the potential of tumor down-staging followed by salvage liver resection in a proportion of patients with initially incurable diseases to become curable, the whole cohort of patients had a median OS of 17 months and PFS of 10 months. The results of our study were in general consistant with the results reported by Finn et al. in a phase 1b single arm study (16) with median OS for patients with uHCC treated with Lenvatinib + pembrolizumab being 22 months, and median PFS being 9.3 months as assessed by mRECIST. The ORR was 46%, and the CR reached 11%. All these results show Lenvatinib-based combination therapies to be a promising treatment for uHCC patients.

In a mouse model, Lenvatinib which targets FGFR 1-4, VEGFR 1-3, PDGF α receptor, RET, and KIT, was shown to reduce the number of monocytes and macrophages while increasing the number of CD8+ T cells. Such effects can enhance the immunomodulatory activity when combined with PD-1 antibody (3, 16). A recent study by Torrens and colleagues found that in addition to inhibiting angiogenesis in tumor tissues, combined Lenvatinib with pembrolizumab exerted distinct immunomodulatory effects by stimulating immunological pathways, decreasing Treg cell infiltration, and blocking TGF-β signaling (17). In a systematic review, combined Lenvatinib with pembrolizumab demonstrated the highest absolute ORR when compared with any first-line treatment for uHCC (18). The combination of Lenvatinib with immune checkpoint inhibitors (ICIs) is promising in treating advanced HCC patients, as recently shown by an analysis of safety and efficacy study conducted by Huang et al. (19). However, in our study, PD-1 when combined with Lenvatinib and TACE did not significantly improve the prognosis of uHCC patients. This suggests that there is no definite correlation among the different types of combination therapy with the therapeutic benefit for patients, and the therapeutic effects may not be improved with more combinations of treatments. In our study, when 22 patients who received Lenvatinib and TACE alone were compared with 20 patients who received in addition PD-1 therapy, and these two groups of patient had similar baseline characteristics with the exception for macrovascular invasion, there was no significant difference in survival outcomes after treatment. Further subgroup analysis of macrovascular invasion was performed. When radiotherapy was added, the median OS of the radiotherapy subgroup on uHCC patients with macrovascular invasion was 13 months, a finding which is similar to the median survival of 12.3 months after radiotherapy reported by Tang et al. (20). All these findings suggest that treatment responses to combined therapy can be related to treatment tolerance by patients, limitations imposed by treatment adverse effects, and/or drug interactions in various combined treatment regimens. Therefore, studies to find out the appropriate combination therapies are important in treating HCC patients with advanced stages of diseases.

Using univariate and multivariate Cox regression analysis, multiple tumor sites was identified as an independent risk factor for post-treatment survival in uHCC patients. To our knowledge, our study is the first to identify multiple tumor sites to be an independent prognostic risk factor in patients with uHCC treated with Lenvatinib-based combination treatments. Yang et al. studied combined TACE with ICIs and TKIs for treatment of uHCC. They found 19 of 31 patients with multiple lesions had a favorable tumor response (21), suggesting that the combined therapy to be a treatment option for patients with uHCC with multiple tumor sites. More researches should be done to find better treatment options for patients with multiple tumor sites which is known to have a poor prognosis.

TACE is the therapy which is commonly used for patients with BCLC intermediate stage HCC, with a high tumor response and a tolerable safety profile (22). Previous studies show that the local anticancer effect of TACE is attributed to its abilities to exacerbate hypoxia in cancer cells (23, 24). Hypoxia in cancer cells triggers production of hypoxia-related factors, which increases VEGF and fibroblast growth factor levels and promotes tumor angiogenesis. In the combined therapy regimen of TACE with Lenvatinib, the better drug can inhibit the kinase activities of VEGF receptors to counteract the adverse effects of TACE, thus resulting in improved anticancer effects. Prior clinical studies indicated that Lenvatinib plus TACE provided survival benefits for patients with uHCC (25, 26). Chen et al. showed that the pembrolizumab-Lenvatinib-TACE sequential therapy prolonged the median PFS and OS in patients with uHCC (27). All these findings suggest that therapy using TACE and Lenvatinib plus pembrolizumab can provide clinical benefits in treating advanced uHCC patients. In our study, some patients treated with TACE and Lenvatinib plus pembrolizumab achieved a prolonged, although not statistically significant, median survival.

The most common adverse events in our study were AST elevation, hypertension, ALT elevation, and decreased appetite, which were similar as reported by the previous phase Ib Study (28). The incidence of grade 3-4 adverse events in our cohort was 24.2%, a figure which is high enough to draw to the attention of clinicians using Lenvatinib-based combination therapies. In general, the incidence of adverse events with combination therapy is higher than with monotherapy. Whether it is local or systemic therapy, the more therapies used, the more are the adverse events, which can have significantly increased impact on the patient’s liver and kidney functions, and can adversely affect the therapeutic effect (29). Therefore, the challenge to use combination therapies in treating uHCC is to find out how to obtain the optional treatment dosages to improve treatment effectiveness with minimal advise effects.

After Lenvatinib-based combination therapies, 15 patients were tumor-downstaged to undergo salvage liver resection with curative intent, making this study to be the first report with the largest sample size of patients with uHCC who underwent down-staging surgery after Lenvatinib-based treatments. While there was no discernible improvement in survival time for those who had surgery compared to those who did not, this provides a novel therapeutic avenue for uHCC patients who were having incurable disease to become potentically curable. The underlying reason why liver resection still needs to be carried out after tumor downstaging is that in a significant proportion of patients, small clusters of cancer cells were left behind which could later lead to HCC recurrence. Excision of the remaining lesions is expected to extend the durations of tumor-free and overall survivals. Qiao et al. performed liver transplantation after treatment with a PD-1 inhibitor and Lenvatinib in 7 patients with HCC. The results suggested promising efficacy with tolerable mortality in the surgically treated patients with uHCC (30). Currently, there are no guidelines or consensus on how and when to carry out salvage surgery for patients after tumor down-staging in patients after treatment with the combination of Lenvatinib and pembrolizumab.

This study has several limitations. First, it is a retrospective study with a small sample size and with a short follow-up. Second, there is a potential risk of selection bias. Third, this study covered a wide range of BCLC stages of uHCC, to include stages A, B, and C. The effectiveness of Lenvatinib is known to vary among the different BCLC stages of HCC. Exclusion of the impact of tumor staging is required in future studies. Fourth, the uHCC patients included in this study were treated with Lenvatinib-based therapy in combination with different other treatments including TACE, PD-1, radiotherapy and so on. These different combinations can result in biases on treatment outcomes, and our conclusions should be interpreted with caution. Fifth, the lack of feedback from biological data in our study warrants further investigation. Finally, only 15 patients in this study had tumor-downstaging after therapy who could fulfill the criteria for salvage surgical resection. The sample size was small, although salvage surgery had a certain impact on prognosis of such patients. A large sample, multicenter, randomized controlled study is needed for further verification.

## Conclusion

Among all the uHCC patients in this study, 58.2% had an objective response rate. For uHCC patients who had an initially poor prognoses, combining anti-angiogenic therapy, immunotherapy, and local treatments have opened up new treatment options. Tumor down-staging followed by salvage liver resection can offer a potential for clinical cure for patients with uHCC who initially is assessed to have incurable diseases.

## Data availability statement

The raw data supporting the conclusions of this article will be made available by the authors, without undue reservation.

## Ethics statement

The studies involving human participants were reviewed and approved by Ethics Committee of Eastern Hepatobiliary Surgery Hospital. The patients/participants provided their written informed consent to participate in this study. Written informed consent was obtained from the individual(s) for the publication of any potentially identifiable images or data included in this article.

## Author contributions

(I) Conception and design: All authors. (II) Administrative support: Z-YP, WL,W-PZ and B-GJ. (III) Provision of study materials or patients: YY, S-XY, F-MG and HL. (IV) Collection and assembly of data: JH, Z-GW and Q-FT. (V) Data analysis and interpretation: All authors. (VI) Manuscript writing: All authors. (VII) Final approval of manuscript: All authors. All authors contributed to the article and approved the submitted version.
